# Comparison of the Postoperative Incidence Rate of Capsular Contracture among Different Breast Implants: A Cumulative Meta-Analysis

**DOI:** 10.1371/journal.pone.0116071

**Published:** 2015-02-13

**Authors:** Xing Liu, Liang Zhou, Fuqiang Pan, Yang Gao, Xi Yuan, Dongli Fan

**Affiliations:** 1 Department of Plastic and Cosmetic Surgery, Xinqiao Hospital, Third Military Medical University, Chongqing, China 400037; 2 Department of Health Statistics, College of Preventive Medicine, Third Military Medical University, Chongqing, China 400038; 3 Department of Plastic and Aesthetic Surgery, Southwest Hospital, Third Military Medical University, Chongqing, China 400038; 4 Department of Plastic and Cosmetic Surgery, Research Institute of Surgery, Daping Hospital, Third Military Medical University, Chongqing, China 400042; Sapienza, University of Rome, School of Medicine and Psycology, ITALY

## Abstract

**Background:**

A large number of clinical studies have reported that the different materials used in breast implants were a possible cause of the different incidence rates of capsular contracture observed in patients after implantation. However, this theory lacks comprehensive support from evidence-based medicine, and considerable controversy remains.

**Objectives:**

In this study, a cumulative systematic review examined breast augmentation that used implants with textured or smooth surfaces to analyze the effects of these two types of implants on the occurrence of postoperative capsular contracture.

**Methods:**

We conducted a comprehensive search of literature databases, including PubMed and EMBASE, for clinical reports on the incidence of capsular contracture after the implantation of breast prostheses. We performed a cumulative meta-analysis on the incidence of capsular contracture in order from small to large sample sizes and conducted subgroup analyses according to the prosthetic material used, the implant pocket placement, the incision type and the duration of follow-up. Relative risks (RR) and 95% confidence intervals (CI) were used as the final pooled statistics.

**Results:**

This meta-analysis included 16 randomized controlled trials (RCTs) and two retrospective studies. The cumulative comparison of textured and smooth breast implants showed statistical significance at 2.13 (95% CI, 1.18-3.86) when the fourth study was entered into the analysis. With the inclusion of more reports, the final results indicated that smooth breast implants were more likely to be associated with capsular contracture, with statistical significance at 3.10 (95% CI, 2.23-4.33). In the subgroup analyses, the subgroups based on implant materials included the silicone implant group and the saline implant group, with significant pooled statistical levels of 4.05 (95% CI, 1.97-8.31) and 3.12 (95% CI, 2.19-4.42), respectively. According to implant pocket placement, a subglandular group and a submuscular group were included in the analyses, and only the subglandular group had a statistically significant pooled result of 3.59 (95% CI, 2.43-5.30). Four subgroups were included in the analyses according to incision type: the inframammary incision group, the periareolar incision group, the transaxillary incision group and the mastectomy incision group. Among these groups, only the pooled results of the inframammary and mastectomy incision groups were statistically significant, at 2.82 (95% CI, 1.30-6.11) and 2.30 (95% CI, 1.17-4.50), respectively. Three follow-up duration subgroups were included in the analyses: the one-year group, the two- to three-year group and the ≥five-year group. These subgroups had statistically significant results of 4.67 (95% CI, 2.35-9.28), 3.42 (95% CI, 2.26-5.16) and 2.71 (95% CI, 1.64-4.49), respectively.

**Conclusion:**

In mammaplasty, the use of textured implants reduces the incidence of postoperative capsular contracture. Differences in implant pocket placement and incision type are also likely to affect the incidence of capsular contracture; however, this conclusion awaits further study.

## Introduction

Breast augmentation with prosthetic implants is a common procedure in plastic and reconstructive surgery. According to a published survey, over 300,000 people received mammaplasty with prosthetic implants in 2011 alone [1]. As a complication of mammaplasty with prosthetic implants, capsular contracture has drawn increasing attention; early reports indicated an incidence rate of over 30% [2–4]. At present, the mechanism of capsular contracture is still unclear, and the process appears to be multifactorial [5]. Some surgeons believe that capsular contracture is a result of cystic fibrosis after long afterprosthetic implantation [6–7]. Based on the characteristics of the prosthesis, Pajkos et al. proposed a microbiological hypothesis that a low-grade bacterial infection or biofilm formation around the implantsmay lead to a robust inflammatory response, and the immune reaction can lead to the secretion of profibrotic cytokines and subsequent contracture [8]. Others researchers have proposed that collagen deposition is also risk factor for the onset of capsular contracture [9–11]. Furthermore, capsular contracture is hypothetically considered a form of hypertrophic scar tissue investing a foreign body or surgically implanted device [12]. Finally, the rupture, deformation or leakage of prosthetic implants is also likely to cause cystic fibrosis and capsular contracture [13–15].

At present, an increasing number of researchers are shifting their research focus to the biocompatibility of bioprostheses [16–23]. The type of implant material used has the most direct impact on a prosthesis’s biocompatibility. In breast reconstruction and augmentation, the implantation of different types of prostheses has been considered a primary factor that affects postoperative capsular contracture [24–25]. With an increasing number of clinical studies, other risk factors have also garnered attention, such as the implant material and surgical approach used [26], but without support from multifactorial evidence-based medicine. Therefore, this study included clinical studies related to capsular contracture after prosthesis implantation and compared the effects of smooth and textured implants on postoperative capsular contracture through cumulative meta-analysis. Based on these findings, comprehensive analyses were performed for other potential risk factors for capsular contracture in the surgical procedure to provide rational support, from the perspective of evidence-based medicine, for reducing the incidence of capsular contracture after prosthetic implantation.

## Methods

### Systematic Literature Search

A literature search was conducted in online databases, including PubMed, EMBASE, SCOPUS, Web of Science, Google Scholar, SinoMed (CBM), the Chinese Medical Citation Index (CMCI/CMCC integrated version), CNKI and the CENTRAL database of the Cochrane Library. The following search strategy was used: ([(Breast reconstruction) OR breast augmentation] AND capsular contracture) AND implants. We performed a manual search of the conference materials at the library of the Third Military Medical University. The search did not include several non-English databases for studies in Spanish and Portuguese, such as LILAC sans SciELO, and it covered the literature published from the year the database was established to December 2013.

### Inclusion Criteria

(1) Participants: Study subjects who received breast augmentation or reconstruction were included.

(2) Intervention: The implantation of smooth implants.

(3) Comparison: The implantation of textured implants.

(4) Outcome: The incidence rates of capsular contracture after implantation.

(5) Study: Literature reports on randomized controlled trials (RCTs) or observational studies were included.

(6) Methodology: I. The Baker grading scale was used as the end diagnostic criteria for disease onset, with Baker grades I and II categorized as “no onset” and Baker grades III and IV categorized as “onset” [27]. II. The determination methods used included palpation and relative applanation tonometry (RAT) [28]. The study group and the control group exhibited consistent baseline data.

### Exclusion Criteria

(1) The studies without control groups were excluded. (2) Duplicate publications, case reports, animal studies, reviews and systematic reviews were excluded. (3) Studies whose study and control group sample sizes differed significantly were excluded.

### Selection and Data Extraction

The included literature was carefully reviewed for information about the first author, publication year, study sample size, study type, end diagnostic indicators and disease conditions of the study and control groups. Two researchers conducted blinded independent data extraction. A third researcher was consulted when there were discrepancies in the data, and agreement was reached after discussion.

### Quality Assessment

Quality assessment was performed using the Cochrane quality assessment criteria [29] for the included RCTs. The retrospective studies were evaluated using the Newcastle-Ottawa quality assessment scale [30]. Two researchers conducted a blinded quality assessment of the included literature. When the researchers’ assessments were discrepant, a third researcher was consulted for the final grading.

### Statistical Analyses

Cumulative analysis was conducted for the extracted data using a pooled random effects model with the sample sizes placed in ascending order. Subgroup meta-analyses were performed according to implant material, implant pocket placement, incision type and follow-up duration. The pooled parameters were relative risk (RR) and 95% confidence interval (CI). Begg’s test and Egger’s test were simultaneously employed to examine publication bias. A sensitivity analysis was completed by converting the pooled results into a fixed effects model. The Stata 11.0 and Revman 5.0 software programs were used in this study.

## Results

### Included Studies

**Fig 1:**
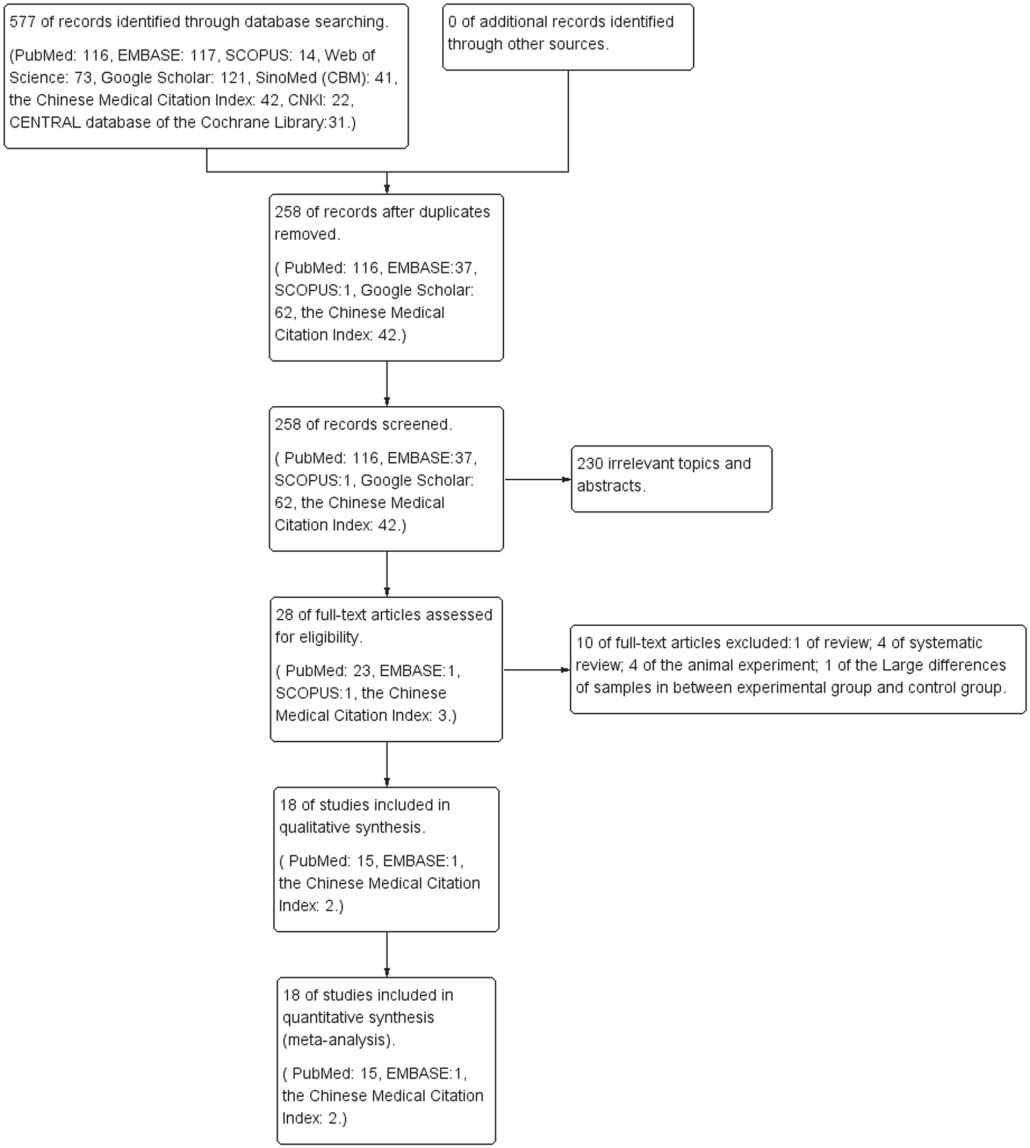
A flow diagram following the PRISMA template.

A total of 577 publications were eventually retrieved from the search. No conference materials were retrieved. After the duplicate publications in the searched electronic library were excluded, 258 publications remained. A total of 230 publications were excluded based on their titles and abstracts, and 28publications remained. Based on the full texts of 28 publications, 2 reviews, 3 reports on systematic reviews, 4 animal studies and 1 report with study and control groups that significantly differed in size were excluded. A total of 18 publications were finally included in this systematic evaluation [31–48]. (Fig. 1)

### Study Characteristics

A total of 18 articles were included in this study, including two retrospective studies and 16 RCTs. The total observed population consisted of 4,486 subjects, with 8,867 implanted breast prostheses and a 100% follow-up rate in 44.44% of these reported studies (8/18). The lowest rate of loss to follow-up was 64%. The 18 studies included three on reconstruction following mastectomy and 15 on breast augmentation. (Table 1)

**Table 1:** Characteristics of included studies.

Author, Year	Age	Intervention methods (/d) (SI^▲^)	Intervention methods (/d) (TI^△^)	Follow-up time & follow-up rate	Diagnostic mode^●^	Patient’s condition	Implant materials	Hematoma	Antibiotics	Implant pocket placement	Incision types	Study design
Coleman, 1991 [31]	N/A	28/48	4/52	1 year & 94%	①	N/A	Silicone implants	1	53	I	Inframammary^◇^	RCT
Lars Hakelius, 1992 [32]	20–45	8/25	0/25	1 year & 100%	②, ③	Bilateral mammary hypoplasia	Silicone implants	3	N/A	I	Inframammary^◇^	RCT
Harlan Pollock, 1993 [33]	N/A	26/196	4/198	1 year & 100%	②	Breast implants	Silicone implants^■^	2	197	I	Inframammary^◇^	Retrospective
Burkhardt, 1994 [34]	20–48	18/45	1/45	1 year & 80%	②	Breast implants	Saline implants^■^	N/A	N/A	I	Periareolar^◇^	RCT
May, J. W., Jr., 1994 [35]	18–54	2/6	0/6	1 year & 100%	②, ③	N/A	Saline implants	N/A	N/A	N/A	Mastectomy^□^	RCT
Burkhardt, B. R., 1995 [36]	42–67	12/62	7/52	2 year & 86.67%	②	Inferior periareolar incision	Saline^■^implants	N/A	N/A	I	Periareolar^◇^	RCT
Thuesen, B., 1995 [37]	22–55	6/9	4/11	2 year & 100%	②	N/A	Silicone implants	N/A	N/A	N/A	Mastectomy^□^	RCT
Asplund, O., 1996 [38]	33–67	17/52	15/67	1 year & 90.02%	①, ③	Bilateral mammary hypoplasia	Silicone implants	1	N/A	II	Inframammary^◇^	RCT
Hammerstad, M., 1996 [39]	19–55	11/46	4/47	3 year & 94.90%	②	Breast cancer	Silicone implants	N/A	0	N/A	Mastectomy^□^	RCT
Hakelius, L., 1997 [40]	25–76	4/8	3/24	5 year & 64%	②	Bilateral mammary hypoplasia	Silicone implants	N/A	N/A	I	N/A	RCT
Malata, C. M., 1997 [41]	20–45	26/44	6/54	3 year & 92.45%	①	N/A	Silicone implants	N/A	N/A	I	N/A	RCT
Tarpila, E., 1997 [42]	N/A	8/21	6/21	1 year & 100%	①	Healthy people	Saline implants	N/A	0	I	N/A	RCT
Collis, N., 2000 [43]	N/A	7/38	2/42	10 year & 75.50%	②	N/A	Silicone implants	N/A	N/A	I	N/A	RCT
Dan, Fagrell 2001 [44]	16–43	6/18	4/18	7.5 year & 90%	②, ③	Healthy people	Saline implants	N/A	N/A	I	Inframammary^◇^	RCT
Wenli Chen, 2005 [45]	25–35	18/508	10/344	1 year & 74.35%	②	Micromastia, bilateral mammary hypoplasia and unilateral breast removal	Silicone implants & Saline implants	N/A	N/A	I	Transaxillary^◇^	RCT
Yanqing Chen, 2005 [46]	19–55	22/204	2/96	2 year & 100%	②	Bilateral mammary hypoplasia and mastatrophy	Silicone implants	N/A	N/A	I	Transaxillary^◇^	RCT
Zhankui Zhu, 2006 [47]	N/A	24/238	4/274	2 year & 100%	②	N/A	Silicone implants	0	N/A	I	Transaxillary^◇^	RCT
Stevens, W. G., 2013 [48]	22–48	214/4157	51/1951	5 year & 100%	②	N/A	Silicone implants	32	3019	I, II	N/A	Retrospective

●① Diagnosed by three doctors

②Diagnosed by the author

③RAT, relative applanation tonometry

■ Iodine

﹡I. Subglandular, II. Submuscular

▲SI, smooth implants

△TI

□incision types of mastectomy

◇Incision types of breast implant; textured implants

RCT, randomized controlled trial.

### Quality Evaluation

The Cochrane quality assessment criteria [29] were adopted to evaluate the 16 included RCTs. Among the included studies, 68.75% (11/18) did not show selection bias, 68.75% (11/18) did not exhibit performance bias, 68.75% (11/18) did not display detection bias, 68.75% (11/18) did not show reporting bias and 18.75% (3/18) did not exhibit other sources of bias. (S1 Fig.)

The Newcastle-Ottawa scale was employed to evaluate the two retrospective studies. Among the *Selection* items, the evaluation results for these two studies were all ≥ three stars, while the evaluation results yielded two stars for the *Comparability* and *Exposure* items. (S1 Table)

### Synthesis of the Results

**Fig 2:**
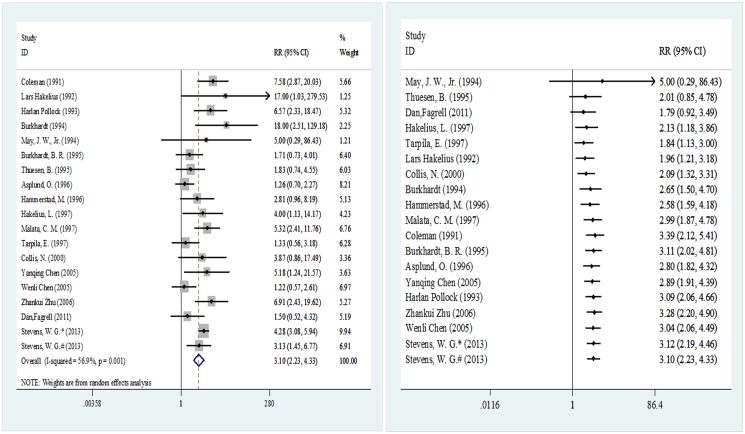
Meta-analysis of overall incidence (random-effects models). Left, standard technique; right, cumulative technique.

As Fig. 2 shows, the overall incidence was obtained based on the 18 clinical studies involving the implantation of 8,458 breast prostheses (5,265 in the smooth implant group and 3,193 in the textured implant group). The meta-analysis results were pooled based on the disease conditions in the last year of the follow-up period. The pooled RR and its 95% CI were 3.10 (2.23–4.33), indicating differences in the probability of capsular contracture after implantation between these two types of prostheses. A cumulative meta-analysis was conducted with the sample sizes in ascending order, and the results indicated that the pooled RR and its 95% CI started to show statistical significance at 2.13 (95% CI 1.18–3.86) from the fourth analyzed study, with gradually stabilizing results afterwards.

**Fig 3:**
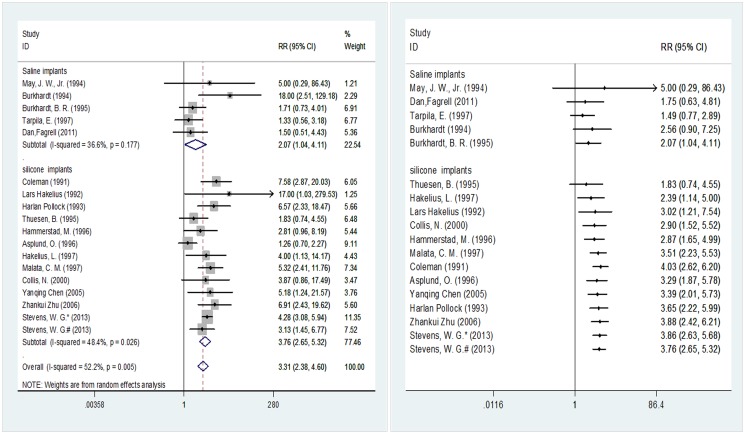
Subgroup meta-analysis according to implant materials (random-effects models). Left, standard technique; right, cumulative technique.

Fig. 3 illustrates the implant materials used in 17 of the included studies involving 8,177 breast prostheses (5,201 in the smooth implant group and 2,968 in the textured implant group). When saline implants were used, the incidence of capsular contracture exhibited a significant difference at 2.07 (95% CI 1.04–4.11) between the two surface types. The pooled results for silicone implants were consistent with those for saline implants, indicating a significant difference at 3.76 (95% CI 2.65–5.32) in the post-implantation incidence of capsular contracture between the two implant surface types. In the cumulative meta-analysis with the sample sizes in ascending order, the pooled RR and its 95% CI started to show statistical significance at 2.07 (95% CI 1.04–4.11) upon the input of the fourth analyzed study [33] when saline implants were used, while the pooled RR and its 95% CI started to show statistical significance at 2.39 (95% CI 1.14–5.00) upon the input of the second analyzed study [37] when silicone implants were used.

**Fig 4:**
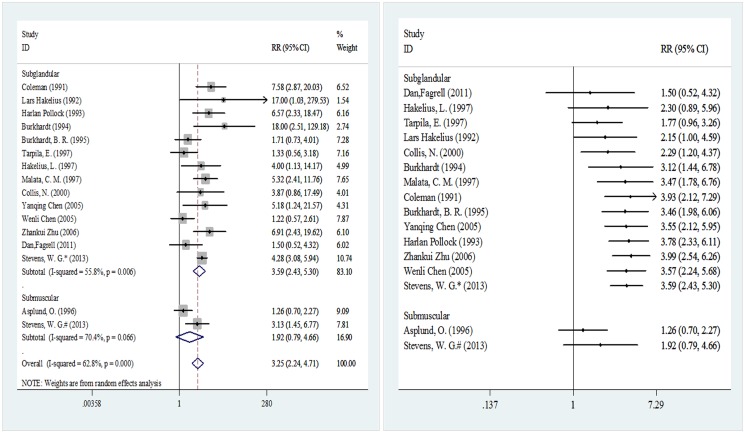
Meta-analysis according to implant pocket placement (random-effects models). Left, standard technique; right, cumulative technique.

Fig. 4 shows the implant pocket placement used in 15 of the included studies involving 7,905 breast prostheses (4,653 in the smooth implant group and 3,252 in the textured implant group). When subglandular implant pocket placement was used, a statistically significant difference was observed in the incidence probability of capsular contracture after implantation between the two surface types of implants, with an RR of 3.59 (95% CI 2.43–5.30). However, when a submuscular implant pocket placement was used, the two implant surface types did not differ in terms of the incidence of capsular contracture, with an RR of 1.92 (0.79–4.66). In the cumulative meta-analysis, the pooled results started to exhibit significance at 2.29 (95% CI 1.20–4.37) upon the input of the fifth analyzed study [43] when subglandular implant pocket placement was used, and the results remained significant until the end of the analysis. However, when submuscular implant pocket placement was employed, the cumulative analysis results were not statistically significant.

**Fig 5:**
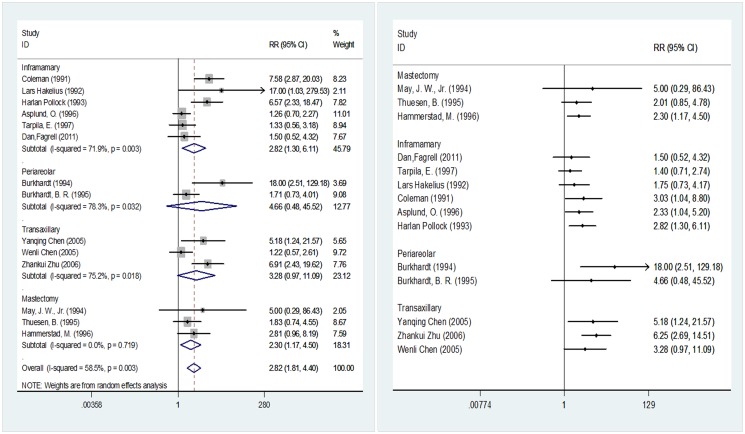
Meta-analysis according to incision type (random-effects models). Left, standard technique; right, cumulative technique.

Fig. 5 shows the incision types used in 14 of the included studies involving 2,711 breast prostheses (1,466 in the smooth implant group and 1,245 in the textured implant group). When the incision type was inframammary or mastectomy, statistically significant differences were detected in the post-implantation probability of capsular contracture between the two surface types of implants, with results of 2.82 (95% CI 1.30–6.11) and 2.30 (95% CI 1.17–4.50), respectively. When the incision type was periareolar or transaxillary, implantation of the two surface types of implants did not yield statistically significant differences in the post-implantation probability of capsular contracture, with results of 4.66 (95% CI 0.48–45.52) and 3.28 (95% CI 0.97–11.09), respectively. In the cumulative meta-analysis, when the incision type was intramammary, the pooled results started to exhibit significance at 3.03 (95% CI 1.04–8.80) upon the input of the fourth analyzed study [31]. When a mastectomy incision was employed, the pooled results started to show significance at 2.30 (95% CI 1.17–4.50) upon the input of the third analyzed study [39]. When the incision type was periareolar, the pooled results began to lose significance at 4.66 (95% CI 0.48–45.52) upon the input of the second analyzed study [36]. When the incision type was transaxillary, the pooled results began to lose significance at 3.28 (95% CI 0.97–11.09) upon the input of the third analyzed study [45].

**Fig 6:**
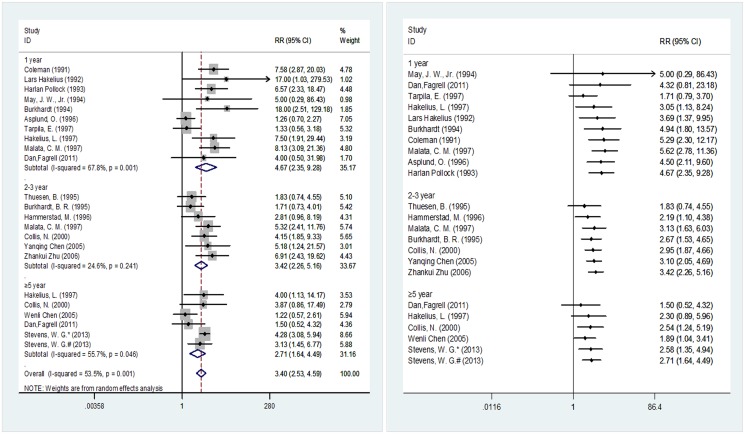
Meta-analysis according to follow-up time (random-effects models). Left, standard technique; right, cumulative technique.

Fig. 6 outlines the follow-up durations of the 18 included studies involving 8,458 breast prostheses (5,265 in the smooth implant group and 3,193 in the textured implant group). In the subgroup analyses, the post-implantation probability of capsular contracture exhibited statistically significant differences between the two implant surface types when the follow-up periods were one, two to three, or ≥ five years, with results of 4.67 (95% CI 2.35–9.28), 3.42 (95% CI 2.26–5.16) and 2.71 (95% CI 1.64–4.49), respectively. In the cumulative meta-analysis with the sample sizes in ascending order, the pooled results began to exhibit significance at 3.05 (95% CI 1.13–8.24) upon the input of the fourth study [40] when the subjects were followed for one year. When the follow-up period was two to three years, the pooled results started to show significance at 2.19 (95% CI 1.10–4.38) upon the input of the second study [39]. When the follow-up period was five years, the pooled results started to show significance at 2.54 (95% CI 1.24–5.19) upon the input of the third study [43].

### Publication Bias and Sensitivity Analysis

Begg’s test and Egger’s test were employed to examine the pooled values from five or more studies. The Begg’s test results indicated no publication bias in any of the analyzed data. The Egger’s test results indicated publication bias (P = 0.049) when the follow-up period was one year; other test results were consistent with those from the Begg’s test (S2 Table). Sensitivity analysis was conducted for the pooled results by converting the pooled model (fixed effects model). The submuscular and periareolar subgroups exhibited large differences in RR values and 95% CIs before and after pooling, indicating instability in the pooled values for these two subgroups. (S2 Table)

## Discussion

### Meta-Analysis Outcomes

In this systematic review, the literature sample size was organized in ascending order to conduct the cumulative meta-analysis. The evaluation compared 18 studies (16 RCTs and 2 retrospective studies) on the incidence of capsular contracture after breast reconstruction and breast augmentation using smooth and textured implants. Based on this comparison, other factors that may affect the occurrence of postoperative capsular contracture were considered on the basis of the following subgroups: implant material, implant pocket placement, incision type and duration of follow-up period. These analyses provided evidence-based practices for such surgeries.

The overall results indicated that smooth implants were more likely to cause capsular contracture after surgery. Some studies demonstrated that the wrinkling of textured implants could prevent capsule formation, thus reducing the incidence of capsular contracture [49–52]. In addition, the differences in the ultra structure of the implanted capsules might account for this finding. Previous studies have indicated that myofibroblasts may play a role in capsular contracture [53]. Specifically, the contractile force produced by these cells on connective fibers, which are arranged in parallel to each other and the implant surface, may generate a resulting vector force perpendicular to the implant, which may result in clinical capsular contracture [54]. Using scanning electron microscopy (SEM), Corrado R et al. observed that multiple layers of fibers surrounded the implants and that these fibers were arranged inlaminae containing parallel fibers [55]. However, in textured implants, the direction of the fibers changed in each layer, which indicated that the arrangement of the fibers made those layers mechanically in effective for the development of a contracting force perpendicular to the implant. As a result, capsular contracture was not easily generated around the textured implants. Also using SEM, Alain MD observed that the adhesive effect was characterizedat the ultrastructural level by a mirror-image tissue response of the periprosthetic capsule to the textured surface of the implant. This indicated that a textured implant with an adhesive effect may represent the best choice for breast reconstruction [56]. Cap BL observed that the ultra structure of smooth implants consisted of a dense, relatively cellular, homogenous, collagenous response, as examined using hematoxylin and eosin (H&E) staining and polarized light of various intensities [57]. Moreover, the dense collagen of smooth implants generates capsular contracture, as textured siliconelacks dense collagen, indicating that smooth implants may develop capsularcontracturemore easily than textured implants. Lance EW et al.proposed that an increasing inflammatory response over time, as seen in textured capsules, might be responsible for a reduction or delay in collagen deposition. Within this setting of continued inflammation, fibrinolyticenzymesmay prevent or delay the development of an organized fibro-collagenous capsule [58].

In conclusion, some studies suggested that the use of textured implants could effectively reduce the occurrence of capsular contracture after breast prosthesis implantation, which is consistent with the results of this study [59–60]. However, there are also cases that are inconsistent with this conclusion. In the cumulative meta-analysis, when the studies were arranged in order of ascending sample size and when only the three studies by May [35], Thuesen [37] and Fagrll [44] were pooled, the results for the two types of prosthesis were not different. However, it is worth noting that the pooled sample size was only 65. The sample was expanded to 101 with addition of Hakelius [40], which started to make the pooled results meaningful, and the results remained meaningful with sample accumulation until all studies were included (Fig. 2). Therefore, a small sample size may be the reason for any false-negative results.

We also noted that in the first three pooled studies, all of the prosthetic materials used were saline implants (Fig. 2). Burkhardt [36] reported that saline implants resulted in better control of postoperative capsular contracture compared with silicone implants. Other studies confirmed these results: with increases in the number of silica particles present, macrophages in the capsule around an implant also increase, causing an aggravation of local inflammation [61–62] that can promote capsular fibrosis and trigger contracture [14, 61]. Silicone implants are commonly used in breast reconstruction and breast augmentation [63]. Although there is relatively strong cohesion between silicone molecules, this cohesion can still fail to prevent implant leakage, leading to inflammation [60]. In Wenli’s study [45],18 out of 320 (5.63%) silicone implants showed hardening compared with 8 out of 532 saline implants (1.50%), which was consistent with fewer complications (including hardening, cracking and leakage) from saline implants compared with silicone implants. Therefore, we performed a subgroup analysis according to different perfusion methods; however, the results indicated that regardless of the perfusion method, smooth and textured implants always differed in terms of the incidence of postoperative capsular contracture (Fig. 3). Nevertheless, the cumulative meta-analysis indicated that when saline implants were used, no differences were observed between pooled results until the addition of the Burkhardt study [36]. The possibility of such false-negative results began to disappear upon sample accumulation. However, we also noted loss to follow-up in the Burkhardt study [36], especially in the textured implants group (Table 1), which could result in the loss of positive cases in the group and would lead to the higher probability of false-positive results in this study. Therefore, whether the use of saline implants can eliminate the difference in postoperative capsular contracture incidence rates between smooth and textured prostheses needs further verification through controlled clinical trials with large sample sizes.

Asplund [38] believed that operative trauma might also affect the incidence of postoperative capsular contracture. Implant pocket placement is one such factor. In a previous meta-analysis, the major surgical approach used in the included population was subglandular, and the final results concluded that textured materials could effectively reduce the incidence of postoperative capsular contracture [60], which is consistent with the results of this study. The cumulative meta-analysis also indicated that sample size is a major factor affecting negative results in early stages (Fig. 4). Some studies suggest that submuscular surgery can effectively control the development of capsular contracture [50, 52, 61]. In our study, no difference was noted between the pooled results of submuscular surgery patients. However, there were only two studies in this subgroup, and the small sample sizes and the appearance of unstable results in the sensitivity analysis (S2 Table) suggest that this result should be further validated.

Incision type is another factor in operative trauma. In previous studies, among 42 periareolar incision cases, a total of four cases of capsular contracture occurred, accounting for 9.5%, while the 338 inframammary incision cases included two cases of capsular contracture, accounting for 0.59%, which led the researchers to conclude that differences in surgical incisions influence postoperative contracture [62]. In our study, four subgroups were created according to incision type: inframammary, periareolar, transaxillary and mastectomy. The results suggested that inframammary incision could not eliminate the difference in the incidence of postoperative capsular contracture between smooth and textured implants. However, we could not conclusively state whether the two implants differed in terms of capsular contracture when periareolar incisions were used (Fig. 5). The inclusion of Haralan Pollock [33] in the inframammary type analysis increased the pooled RR value and 95% CI. Because this study is a retrospective study, this inclusion will introduce certain biases and needs to be further examined. The 95% CIs of the inframammary and periareolar subgroups overlapped, so we could not conclude that these groups differed in their control of the incidence of postoperative capsular contracture; this finding is inconsistent with the conclusion of Wiener, TC [62]. In the transaxillary subgroup, the pooled result was not statistically significant, and the main heterogeneity arose from the Wenli Chen study [45]. We believe that the differences in these results may not be related to the different incision types. In the study by Wenli Chen [45], saline and silicone implants and two different perfusion methods were evaluated in a large sample. (Table 1) The use of saline implants may be the main reason why no difference was noted between the pooled results. Mastectomy incisions are mainly performed on mastectomy patients; Fig. 5 shows that after pooling three studies with no statistical significance [35, 37, 39]. It is noteworthy thatthe subjects inthese threestudieswereallpatients undergoingbreast reconstructionsurgery, whereas the subjects in the other included studieswerepatients undergoing breast augmentation. According to the literature, breast reconstruction is mainly applied in breast reconstructionafter mastectomy [64]. Clinically, mastectomy is mainly performed for the treatment of breast tumors or breast cancer [39], Poland’s syndrome [65], andbreast tissuelosscaused byinfectionorburns [66]. Although such treatment can be achievedbymastectomy, the surgical traumaandresidualcancerous orinflammatory tissueresulting from this procedure canaffect the normalbreast tissue, resulting ina less healthy breastafterreconstruction. In particular, pathologicaldamagetobreast tissue [67]ordeepbreastabscessescaused by inflammation [68]mayincreasethe incidence ofpostoperative capsularcontractureafter breast reconstruction. However, the subjects undergoing breast augmentation in our study were normalhealthy individualswho received reconstruction only to improve the cosmetic result. Thus, the results ofthe meta-analysismayhave been affected bydifferences inthe situationof the breast between these two groups of patients. Moreover, due tothe lackof anobjectiveassessment index for thebreastandthe correlation between the basic breastsituationandcapsular contracture, the comparisonofthe breastdifferences betweenthese two groups of patientscannot beconsidered significant. The pooled results start to become statistically significant, which could reflect the small sample sizes of the individual studies. Therefore, we believe that the use of textured implants in mastectomy is meaningful for controlling the occurrence of postoperative capsular contracture.

We performed a subgroup analysis according to the duration of follow-up. In this analysis, the comparison of smooth and textured implants revealed that both types could reduce the incidence of postoperative capsular contracture, a conclusion that is consistent with previous reports. Among the ≥e year subgroup, Stevens, W.G. [48] is a retrospective study, which might cause inconsistency in the pooled results. However, because Stevens, W.G. [48] has a relatively large sample size and good evaluation result (S2 Table), we can consider the pooled result to be reliable.(Fig. 6)

### Limitations

Among the included papers, the research subjects were breast reconstruction patients in three papers [35, 37, 39] and were research subjects and breast augmentation patients in all other papers. There were certain differences in these two types of patients’ basic information, such as immunologic status, skin conditions, previous resection of subcutaneous and glandular tissue, which might introduce heterogeneity in the pooled results. According to the quality evaluation results, the quality of the included Chinese literature [45–47] was relatively low, particularly concerning the lack of randomized settings and blinding methods in the experimental procedures. Compared with the Chinese literature, the quality of the English literature was relatively high, but there were still ambiguities in the reports in terms of reporting bias and other types of bias. It is worth noting that loss to follow-up existed in eight papers included in this study, but none of those studies used the intention-to-treat (ITT) method to process the number of patients lost to follow-up. For example, in the study by Burkhardt, BR, 1995 [36], the missing samples came from the textured implants group; thus, the probability of a type 1 error of the pooled result of the saline implants subgroup in Fig. 2 would increase if all of the missing cases were positive. In addition, studies in the literature [38, 69–71] indicatethat bleeding, infection and the use of antibiotics during surgery are all factors associated with postoperative capsular contracture, but only 33.33% (6/18) of the included literature in this systematic review provides relevant information (Table 1), which makes further analysis impossible. Additionally, two retrospective studies whose reasoning levels were lower relative to the RCTs were included in this study. However, the quality evaluation of the retrospective studies was better, and the sample sizes were large; thus, these differences likely did not impact the pooled results.

## Conclusion

This study suggests that relative to smooth implants, textured implants can reduce the probability of capsular contracture after breast implantation. Small sample size was one of the factors responsible for negative conclusions in experimental results. By improving surgical procedures and selecting optimal perfusion materials and surgical types, the incidence of postoperative capsular contracture can be reduced.

## Supporting Information

S1 Fig Risk of bias graph& risk of bias summary. (TIF)

S1 Table The result of Newcastle-Ottawa scale (NOS). Selection: 1. Representativeness of the exposed cohort. 2. Selection of the non-exposed cohort. 3. Ascertainment of exposure.4. Demonstration that the outcome of interest was not present at the beginning of the study; Comparability:1. Comparability of the cohorts on the basis of the design or analysis; Exposure:1. Assessment of the outcome. 2. Was the follow-up long enough for outcomes to occur? 3. Adequacy of the follow-up of the cohorts.
(DOC)

S2 Table The result of Publication Bias & Sensitivity analysis. *Statistical significance; N/A: not applicable.
(DOC)

S1 File PRISMA 2009 Checklist. (DOC)
