# Supplementary figures and images for: Comparison of the Postoperative Incidence Rate of Capsular Contracture among Different Breast Implants: A Cumulative Meta-Analysis
Source: PLoS One. 2015 Feb 13;10(2):e0116071. doi: 10.1371/journal.pone.0116071 (PMC4332657; doi:10.1371/journal.pone.0116071)

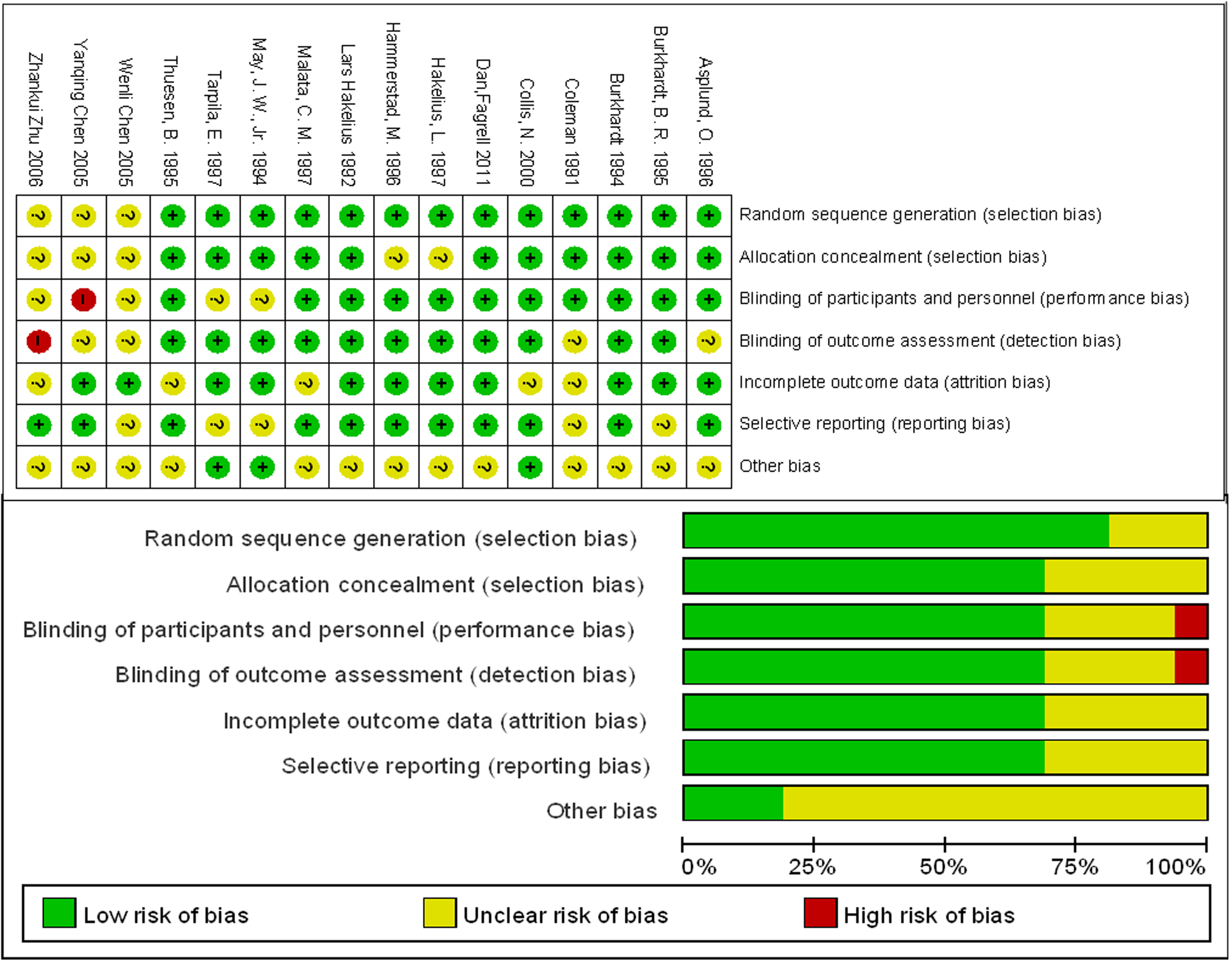

Supplement: S1 Fig — (TIF) [file pone.0116071.s001.tif]
