# Supplementary material for: Comparison of the Postoperative Incidence Rate of Capsular Contracture among Different Breast Implants: A Cumulative Meta-Analysis
Source: PLoS One. 2015 Feb 13;10(2):e0116071. doi: 10.1371/journal.pone.0116071 (PMC4332657; doi:10.1371/journal.pone.0116071)
Supplement: S1 Table — Selection: 1. Representativeness of the exposed cohort. 2. Selection of the non-exposed cohort. 3. Ascertainment of exposure.4. Demonstration that the outcome of interest was not present at the beginning of the study; Comparability:1. Comparability of the cohorts on the basis of the design or analysis; Exposure:1. Assessment of the outcome. 2. Was the follow-up long enough for outcomes to occur? 3. Adequacy of the follow-up of the cohorts. (DOC) [file pone.0116071.s002.doc]

Table S1. The result of Newcastle-Ottawa scale (NOS)

| First author | Published year | Selection | Comparability | Exposure |
| --- | --- | --- | --- | --- |
| Harlan Pollock | 1993 | ★★★★ | ★★ | ★★ |
| Stevens, W. G. | 2013 | ★★★ | ★★ | ★★ |

Selection: 1. Representativeness of the exposed cohort. 2. Selection of the non-exposed cohort. 3. Ascertainment of exposure. 4. Demonstration that the outcome of interest was not present at the beginning of the study; Comparability: 1. Comparability of the cohorts on the basis of the design or analysis; Exposure: 1. Assessment of the outcome. 2. Was the follow-up long enough for outcomes to occur? 3. Adequacy of the follow-up of the cohorts.
