# Supplementary material for: Comparison of the Postoperative Incidence Rate of Capsular Contracture among Different Breast Implants: A Cumulative Meta-Analysis
Source: PLoS One. 2015 Feb 13;10(2):e0116071. doi: 10.1371/journal.pone.0116071 (PMC4332657; doi:10.1371/journal.pone.0116071)
Supplement: S2 Table — *Statistical significance; N/A: not applicable. (DOC) [file pone.0116071.s003.doc]

Table S2. The result of Publication Bias & Sensitivity analysis

| **Object** | Begg’s test (Z) | Begg’s test (*Pr* ) | Eegg’s test (*t*) | Eegg’s test (*P*) | Random effects model (RR and 95%CI) | Fixed effects model (RR and 95%CI) |
| --- | --- | --- | --- | --- | --- | --- |
| **Overall incidence** | 0.99 | 0.322 | 0.24 | 0.815 | 3.10 (95%CI 2.23～4.33) | 3.52 (95%CI 2.92～4.25) |
| **The implant materials** |  |  |  |  | 3.31 (95%CI 2.38～4.60) | 3.31 (95%CI 2.38～4.60) |
| Silastic implants | 0.60 | 0.548 | 2.52 | 0.053 | 4.05 (95%CI 1.97～8.31) | 4.05 (95%CI 1.97～8.31) |
| Silicone implants | 0.47 | 0.640 | -0.57 | 0.583 | 3.12 (95%CI 2.19～4.42) | 3.12 (95%CI 2.19～4.42) |
| **Implant pocket placemen**t |  |  |  |  | 3.25 (95%CI 2.24～4.71) | 3.59 (95%CI 2.96～4.36) |
| Subglandular | 0.77 | 0.443 | 0.29 | 0.777 | 3.59 (95%CI 2.43～5.30) | 4.01 (95%CI 3.24～4.96) |
| Submuscular | N/A | N/A | N/A | N/A | 1.92 (95%CI 0.79～4.66) | 2.07 (95%CI 1.28～3.36) |
| **Incision types** |  |  |  |  | 2.82 (95%CI 1.17～4. 50) | 2.99 (95%CI 2.31～3.87) |
| Inframamary | 0.75 | 0.452 | 1.64 | 0.177 | 2.82 (95%CI 1.30～6.11) | 2.94 (95%CI 2.06～4.21) |
| Periareolar | N/A | N/A | N/A | N/A | 4.66 (95%CI 0.48～45.52) | 3.75 (95%CI 1.79～7.84) |
| Transaxillary | N/A | N/A | N/A | N/A | 3.28 (95%CI 0.97～11.09) | 2.96 (95%CI 1.74～5.04) |
| Mastectomy | N/A | N/A | N/A | N/A | 2.30 (95%CI 1.17～4.50) | 2.51 (95%CI 1.24～5.06) |
| **The follow-up time** |  |  |  |  | 3.40 (95%CI 2.53～4.29) | 3.74 (95%CI 3.13～4.46) |
| 1 year | 0.18 | 0.858 | 2.31 | 0.049* | 4.67 (95%CI 2.35～9.28) | 4.35 (95%CI 3.13～6.05) |
| 2-3 year | 0.00 | 1.000 | 0.39 | 0.714 | 3.42 (95%CI 2.26～5.16) | 3.80 (95%CI 2.66～5.43) |
| ≥5 year | 0.00 | 1.000 | -1.23 | 0.287 | 2.71 (95%CI 1.64～4.49) | 3.38 (95%CI 2.61～4.38) |

*Statistical significance; N/A: not applicable.
