# Supplementary material for: Comparison of the Postoperative Incidence Rate of Capsular Contracture among Different Breast Implants: A Cumulative Meta-Analysis
Source: PLoS One. 2015 Feb 13;10(2):e0116071. doi: 10.1371/journal.pone.0116071 (PMC4332657; doi:10.1371/journal.pone.0116071)
Supplement: S1 File — (DOC) [file pone.0116071.s004.doc]

PRISMA 2009 Checklist

| **Section/topic** | **#** | **Checklist item** | **Reported on page #** |
| --- | --- | --- | --- |
| TITLE | | | |
| Title | 1 | Comparison of the Postoperative Incidence Rate of Capsular Contracture among Different Breast Implants: A Cumulative Meta-Analysis | Page 1 |
| ABSTRACT | | | |
| Structured summary | 2 | Background: A large number of clinical studies have reported that breast implants of different materials were a possible cause for the different incidence rates of capsular contracture observed in patients after implantation. However, this notion lacks comprehensive support from evidence-based medicine, and considerable controversy still remains.  Objectives: In this study, a cumulative systematic review was performed on breast augmentation using implants with textured or smooth surfaces to analyze the effects of these two types of implants on the occurrence of postoperative capsular contracture.  Methods: A comprehensive search was conducted in literature databases, including PubMed and EMBASE, for clinical reports on the incidence of capsular contracture after implantation of breast prostheses. We performed a cumulative meta-analysis on the incidence of capsular contracture in the order from small to large sample sizes and conducted subgroup analyses according to prosthetic material, implant pocket placement, incision type and duration of follow-up period. Relative risk (RR) and 95% confidence interval (CI) were used as the final pooled statistics.  Results: This meta-analysis included 16 randomized controlled trials (RCTs) and 2 retrospective studies. The cumulative comparison of textured and smooth breast implants showed statistical significance at 2.13 (95% CI, 1.18-3.86) when the fourth study was entered into the analysis. With the inclusion of more reports, the final results indicated that smooth breast implants were more likely to be associated with capsular contracture, with statistical significance at 3.10 (95% CI, 2.23-4.33). In the subgroup analyses, the subgroups based on implant materials included the silicone implant group and the saline implant group, with significant pooled statistical levels at 4.05 (95% CI, 1.97-8.31) and 3.12 (95% CI, 2.19-4.42), respectively. According to implant pocket placement, subglandar group and submuscular group were included in the analyses, and only the subglandar group had a statistically significant pooled result at 3.59 (95% CI, 2.43～5.30). Four subgroups were included in the analyses according to incision type: the inframammary incision group, the periareolar incision group, the transaxillary incision group and the mastectomy incision group. Among these groups, only the pooled results of the inframammary and mastectomy incision groups had statistically significant results, at 2.82 (95% CI, 1.30-6.11) and 2.30 (95% CI, 1.17-4.50), respectively. Based on the follow-up period duration, three subgroups were included in the analyses: the 1-year group, the 2-3-year group and the≥5-year group. These subgroups had statistically significant results at 4.67 (95% CI, 2.35-9.28), 3.42 (95% CI, 2.26-5.16) and 2.71 (95% CI, 1.64-4.49), respectively.  Conclusion: In mammaplasty, the use of textured implants reduced the incidence of postoperative capsular contracture. Differences in implant pocket placement and incision type are also likely to affect the incidence of capsular contracture, which awaits further study. | Page 3 |
| INTRODUCTION | | | |
| Rationale | 3 | Therefore, this study included clinical studies related to capsular contracture after prosthesis implantation and compared the impacts of smooth and texture implants on postoperative capsular contracture through cumulative meta-analysis. | Page 4 |
| Objectives | 4 | Based on these findings, comprehensive analyses were performed for other potential risk factors in the surgical procedure for capsular contracture to provide rational support, from the perspective of evidence-based medicine, for how to reduce the incidence of capsular contracture after prosthetic implantation. | Page 4 |
| METHODS | | | |
| Protocol and registration | 5 | No. |  |
| Eligibility criteria | 6 | **Inclusion Criteria**  (1) Literature on randomized controlled trials (RCTs) or observational studies was included. (2) Study subjects who received breast augmentation or reconstruction were included. (3) The incidence rates of capsular contracture after implantation was compared between smooth implants and textured implants. (4) The Baker Grade scale was used as the end diagnostic criteria for disease onset, with Baker grades I and II as no onset and Baker grades III and IV as onset. (5) The determination methods used included palpation and relative applanation tonometry (RAT). (6) The subjects had consistent baseline data between the study group and the control group.  **Exclusion Criteria**  (1) Studies without control groups were excluded. (2) Duplicate publications, animal studies, research reviews and systematic evaluations were excluded. (3) Studies with significantly different sample sizes between the study and control groups were excluded. | Page 4 |
| Information sources | 7 | A literature search was conducted in online databases, including PubMed, EMBASE, SCOPUS, Web of Science, Google Scholar, SinoMed (CBM), the Chinese Medical Citation Index (CMCI/CMCC integrated version), CNKI and the CENTRAL database of the Cochrane Library. | Page 4-5. |
| Search | 8 | The search strategy was as follows: ([(Breast reconstruction) OR Breast augmentation] AND Capsular contracture) AND implants. Meanwhile, a manual search was performed within the conference materials held at the library of the Third Military Medical University. The search covered the literature published from the year when a database was set up to December 2013. | Page 4. |
| Study selection | 9 | A total of 577 publications were eventually retrieved from the search. No conference materials were retrieved. After the duplicate publications in the searched electronic library were excluded, 289 publications remained. A total of 230 publications were excluded based on their titles, and 28 publications remained. Based on abstracts and full text, two reviews, three reports on systematic evaluations, four animal studies and one report with study and control groups that significantly differed in size were excluded. A total of 18 publications were finally included in this systematic evaluation | Page 6. |
| Data collection process | 10 | A total of 577 publications were eventually retrieved from the search. No conference materials were retrieved. After the duplicate publications in the searched electronic library were excluded, 289 publications remained. A total of 230 publications were excluded based on their titles, and 28 publications remained. Based on abstracts and full text, two reviews, three reports on systematic evaluations, four animal studies and one report with study and control groups that significantly differed in size were excluded. A total of 18 publications were finally included in this systematic evaluation. | Page 6. |
| Data items | 11 | As shown in Figure 2, the overall incidence was obtained based on the 18 clinical studies involving implantation of 8,458 breast prostheses (5,265 in the smooth implant group and 3,193 in the textured implant group).  Figure 3 illustrates the implant materials used in 17 of the included studies, involving 8,177 breast prostheses (5,201 in the smooth implant group and 2,968 in the textured implant group).  Figure 4 shows the implant pocket placement used in 15 of the included studies, involving 7,905 breast prostheses (4,653 in the smooth implant group and 3,252 in the textured implant group).  Figure 5 shows the incision types used in 14 of the included studies, involving 2,711 breast prostheses (1,466 in the smooth implant group and 1,245 in the textured implant group). | Page 6-7. |
| Risk of bias in individual  studies | 12 | Quality assessment was performed using the Cochrane quality assessment criteria for the included RCTs. The retrospective studies were evaluated using the Newcastle-Ottawa quality assessment scale. Two researchers conducted blinded quality assessment for the included literature. When discrepancy existed for a study, a third researcher was consulted for the final grading. | Page 5. |
| Summary measures | 13 | Cumulative analysis was conducted for the extracted data using a pooled random effects model, with the sample sizes in ascending order. Subgroup meta-analyses were performed according to implant material, implant pocket placement, incision type and duration of follow-up period. The pooled parameters were relative risk (RR) and 95% confidence interval (CI). Begg’s test and Eeeg’s test were simultaneously employed to examine publication bias. Sensitivity analysis was completed through converting the pooled results into a fixed effects model. The software Stata 11.0 and Rveman 5.0 were used in this study. | Page 5-6. |
| Synthesis of results | 14 | Cumulative analysis was conducted for the extracted data using a pooled random effects model, with the sample sizes in ascending order. Subgroup meta-analyses were performed according to implant material, implant pocket placement, incision type and duration of follow-up period. The pooled parameters were relative risk (RR) and 95% confidence interval (CI). | Page 5. |
| Risk of bias across studies | 15 | The retrospective studies were evaluated using the Newcastle-Ottawa quality assessment scale. Two researchers conducted blinded quality assessment for the included literature. When discrepancy existed for a study, a third researcher was consulted for the final grading. | Page 5. |
| Additional analyses | 16 | Begg’s test and Eeeg’s test were simultaneously employed to examine publication bias. Sensitivity analysis was completed through converting the pooled results into a fixed effects model. | Page 5-6. |
| RESULTS | | | |
| Study selection | 17 | A total of 541 publications were eventually retrieved from the search, with no conference materials. After excluding the duplicate publications entered into the searched electronic library, 257 publications remained. A total of 230 publications were excluded based on their titles, and 27 publications remained. Based on the abstract and the full text, 1 review, 3 reports on systematic evaluations, 4 reports on animal studies and 1 report with study and control groups of significantly different sample sizes were excluded. A total of 18 publications were finally included in this systematic evaluation. | Page 6. |
| Study characteristics | 18 | A total of 18 articles were included in this study, including 2 retrospective studies and 16 RCTs. The total observed population consisted of 4,486 subjects, with 8,867 implanted breast prostheses and a 100% follow-up rate in 44.44% of these reported studies (8/18). The lowest rate of loss to follow-up was 64%. The 18 studies included 3 on reconstruction following mastectomy and 15 on breast augmentation. | Page 6. |
| Risk of bias within studies | 19 | The Cochrane quality assessment criteria were adopted to evaluate the included 16 RCTs. Among the included studies, 68.75% (11/18) did not show selection bias, 68.75% (11/18) did not exhibit performance bias, 68.75% (11/18) did not display detection bias, 68.75% (11/18) did not show reporting bias and 18.75% (3/18) did not exhibit other sources of bias.  The Newcastle-Ottawa scale was employed in the evaluation of the two retrospective studies. Among the *Selection* items, the evaluation results of these two studies were all ≥3 stars, while the evaluation results were all 2 stars for *Comparability* and *Exposure* items. | Page 6. |
| Results of individual studies | 20 | N/A. | N/A. |
| Synthesis of results | 21 | Please see Figure 2-6. | Figure 2-6 |
| Risk of bias across study | 22 | Begg’s test and Eegg’s test were employed to examine the pooled values from 5 or more studies. The Begg’s test results indicated no publication bias in any of the analyzed data. The Eegg’s test results indicated publication bias (P = 0.049) when the follow-up period was 1 year, with other test results consistent with those from the Begg’s test (Table S2). Sensitivity analysis was conducted for the pooled results through converting the pooled model (fixed effects model). The submuscular and periareolar subgroups exhibited large differences in RR values and 95% CIs before and after pooling, indicating instability in the pooled values in these two subgroups. | Figure 8. |
| Additional analysis | 23 | Sensitivity analyses. | Figure 2-6 |
| DISCUSSION | | | |
| Summary of evidence | 24 | In this systematic review, the literature sample size was organized in ascending order to conduct the cumulative meta-analysis. For the evaluation, 18 studies (16 RCTs and 2 retrospective studies) of the incidence of capsular contracture after breast reconstruction and breast augmentation using smooth and textured implants were compared. On the basis of this comparison, other factors affecting the possible occurrence of postoperative capsular contracture were considered according to the following subgroups: implant material, implant pocket placement, incision type and duration of follow-up period. These analyses provided evidence-based medicine for such surgeries. | Page 8. |
| Limitations | 25 | According to quality evaluation results, the quality of the included Chinese literature [43] [44] [45] was relatively low, particularly concerning the lack of application of randomized settings and blinding methods in the experimental procedures. Compared with the Chinese literature, the quality of English literature was relatively high, but there were still ambiguities in the reports in terms of reporting bias and other types of bias. It is worth noting that loss to follow-up existed in 8 papers included in this study, but none of those studies used the intention-to-treat (ITT) method for processing the number of patients lost to follow-up. For example, in the study of Burkhardt, BR, 1995 [34], the missing samples were from the textured implants group; thus, the probability of type 1 error of the pooled result of the saline implants subgroup in Figure 2 would increase if all of the missing cases were positive. In addition, studies in the literature indicate [36] [61-63] that bleeding, infection and the use of antibiotics during surgery are all factors associated with postoperative capsular contracture, but only 33.33% (6/18) of the included literature in this systematic review provides relevant information (Table 1), which makes further analysis impossible. Additionally, two retrospective studies were included in this study, whose reasoning levels were lower relative to RCTs. However, the quality evaluation of the retrospective studies was better, and the sample sizes were large; thus, these differences likely did not impact the pooled results. | Page 10-11. |
| Conclusions | 26 | This study suggests that relative to smooth implants, textured implants can reduce the probability of the incidence of capsular contracture after breast implantation. Small sample size was one of the factors responsible for negative conclusions in experimental results. Improving surgical procedures and selecting optimal perfusion materials and surgical types could reduce the incidence of postoperative capsular contracture. | Page 11. |
| FUNDING | | | |
| Funding | 27 | This work is partly supported by Grants from the National Natural Science Foundation of China (81071574, 81372075). | N/A |
